# Assistive technology use and human rights enjoyment: a cross-sectional study in Bangladesh

**DOI:** 10.1186/1472-698X-12-18

**Published:** 2012-09-19

**Authors:** Johan Borg, Stig Larsson, Per-Olof Östergren, ASM Atiqur Rahman, Nazmul Bari, AHM Noman Khan

**Affiliations:** 1 Department of Health Sciences, Lund University, Malmö, Sweden; 2 Social Medicine and Global Health, Lund University, Malmö, Sweden; 3 Institute of Social Welfare and Research, University of Dhaka, Dhaka, Bangladesh; 4 Centre for Disability in Development, Savar, Bangladesh

## Abstract

**Background:**

About half a billion people with disabilities in developing countries have limited access to assistive technology. The Convention on the Rights of persons with Disabilities requires governments to take measures to ensure provision of such technologies. To guide implementation of these measures there is a need for understanding health outcomes from a human rights perspective. The objective of this study was therefore to explore the relation between assistive technology use and enjoyment of human rights in a low-income country.

**Methods:**

Data was collected in eight districts of Bangladesh through interviews of people with hearing impairments using and not using hearings aids, and people with ambulatory impairments using and not using manual wheelchairs (N = 583). Using logistic regression, self-reported outcomes on standard of living, health, education, work, receiving information and movement were analyzed.

**Results:**

The adjusted likelihood of reporting greater enjoyment of human rights was significantly higher among people using hearing aids compared to non-users for all outcomes except working status. Compared to non-users, users of wheelchairs reported a significantly higher adjusted likelihood of good ambulatory performance and a significantly lower adjusted likelihood of reporting a positive working status. Further analyses indicated that physical accessibility to working places and duration of wheelchair use had a statistically significant impact on the likelihood of reporting positive work outcomes.

**Conclusions:**

The findings support the notion that assistive technology use increases the likelihood of human rights enjoyment, particularly hearing aid use. Physical accessibility should always be addressed in wheelchair provision.

## Background

Is there a positive relation between use of assistive technology and enjoyment of basic human rights in low-income countries? Although the Convention of the Rights of Persons with Disabilities (CRPD) and the Standard Rules on the Equalization of Opportunities for Persons with Disabilities (Standard Rules) require assistive technology interventions to facilitate full enjoyment of human rights [1,2], little empirical data is available to support such claims in low-income countries. Indications of certain benefits in areas such as health, mobility and education have been published [3-5].

The lack of knowledge about human rights effects of assistive technology interventions in low-income countries is not unique to this field. Attention has recently been drawn to the need for understanding, measuring and improving rehabilitation outcomes from a human rights perspective, where rights themselves might be valued outcomes [6]. Out of a concern that measures of specialist services quality became increasingly bureaucratic and devoid of meaningful content, CRPD based human rights indicators have been suggested [7].

The human rights were first formulated in the Universal Declaration of Human Rights (UDHR) from 1948 [8]. This was followed by two covenants that define civil, political, economic, social and cultural rights [9,10]. To provide guidance on how to ensure specific rights, or protect the rights of specific groups of people, various conventions have been adopted; the CRPD being one of them. With few exceptions, people in low-income countries enjoy human rights to a much less extent than people living in countries with richer economies, particularly with regards to their standard of living, health, education and work [11]. Disability often enlarges this gap resulting in people with disabilities being amongst the most marginalized in every society, particularly in low-income countries [12,13].

It is estimated that about half a billion people with disabilities live in developing countries and that only 5-15% of those who need have access to assistive technologies [14,15]. In some African countries, the largest gap between needed and received disability related services was with regard to assistive technology [16]. Factors limiting access to assistive technology includes lack of products, skilled personnel, suitable infrastructure and financial means [17]. The lack of assistive technologies is aggravated by the fact that the need for associated services is seldom considered [18]. It is therefore promising that the CRPD requires governments to provide affordable assistive technologies along with necessary services in order to ensure the full and equal enjoyment of all human rights of people with disabilities [17]. To implement these measures, there is a need for developing appropriate, evidence-based strategies for provision of these technologies and services. However, prior to this, we need empirical knowledge on what role assistive technologies plays in enabling their users to enjoy human rights in order to set priorities in provision. The objective of this study was therefore to contribute to filling the current gap of empiric evidence by exploring the relation between assistive technology use and enjoyment of basic human rights in a low-income country.

## Methods

### Context

Data for this study was collected in Bangladesh, which has an estimated population of about 164 million people living on 147 thousand square kilometres of land. In 2009, it ranked 146 out of 182 countries on the Human Development Index. The life expectancy at birth was 65.7 years, the adult literacy rate was 53.5%, and the GDP per capita was PPP US$ 1,241. About 40% of the population live below the national poverty line and about 50% live on less than $1.25 a day [19,20].

A recent study indicates a disability prevalence rate in Bangladesh of about 6% [21], which corresponds to approximately 10 million people. Disability has been reported to have a devastating effect on quality of life, particularly on educational attainment and employment [22]. In 2001, Bangladesh adopted the Persons with Disability Welfare Act. This was followed by the ratification of the CRPD in 2007 and its Optional Protocol in 2008. Thus, in principle, the country supports equal rights and opportunities for people with disabilities. However, for most of them, these rights have not been realized as their access to development programmes, social benefits, and health and rehabilitation services is limited [23,24]. To promote the rights of people with disabilities, 46 Focal Points have been established in different ministries and departments, and a committee has been set up to monitor the implementation of the CRPD. In addition, a Disability Rights Watch Group has been formed with representatives from civil society and the Parliamentarians’ Caucus on Disability. Progress has been made in developing a new law for persons with disability based on human rights.

According to the World Health Organization (WHO), an estimated 1.6 million people in Bangladesh would need a wheelchair and about 0.8 million people would need an orthotic device [25,26]. Further, based on the situation in countries like Indonesia and Nigeria, an estimated five million or more Bangladeshis would benefit from using a hearing aid [27]. Less is known about other types of assistive technology. Although there has been some government, non-government and private initiatives to make assistive technology accessible, the needs for assistive technology are far from being met [28,29]. Apart from services being physically, geographically and economically inaccessible, lack of trained personnel is another reason for this gap. This may be exemplified by comparing the current some 50 orthopaedic technicians working in Bangladesh with a required number of 5,000 personnel trained at different levels to conform to WHO recommendations [26].

### Sample

The sample in this study was derived from a survey which aimed at exploring the relationships between the use of assistive technology and the enjoyment of human rights and the economic situation in a population of people with hearing and ambulatory impairments living in Bangladesh. The survey was cross-sectional using an interviewer-administered structured questionnaire to collect quantitative data. The inclusion criteria included people with hearing impairments using or not using hearing aids or people with ambulatory impairments using or not using manual wheelchairs in the age-group 15–55 years. To achieve a statistical power of 0.8 when using logistic regression and an effect size corresponding to an odds ratio (OR) of 2.0, a sample size of 222 is required. (see Additional file 1: Appendix 1 for calculation details.)

Due to lack of government registers of people with disabilities in general, and users of assistive technology in particular, the non-government organization Centre for Disability in Development (CDD) was contacted in order to find eligible respondents. CDD is the largest disability oriented, national resource and training centre in Bangladesh with over 300 partner organizations across the country, through which it has access to locally maintained registers of people with disabilities, including users of assistive technology. The way people had been included in the registers varied between and within the organizations. Main methods to identify people with disabilities were: during community meetings attended by people with disabilities, information provided by community people, home visits based on information from local people and authorities, people with disabilities voluntarily approaching the organizations, people with disabilities referring other people with disabilities, and surveys. The proportion of people being recruited by what method is unknown. However, there was no obvious difference in the chance to be included in those registers because of use of assistive technology or not.

Sample representation from four typical areas of Bangladesh was sought; in and around the capital Dhaka, general countryside, areas prone to flooding, and hilly areas. Based on minimizing the number of involved organizations in the selected areas in order to achieve a target sample size of 300 people with each impairment, eight organizations were selected for collection of data from people with ambulatory impairments and ten =organizations were selected for collection of data from people with hearing impairments in eight districts (Bogra, Chittagong, Dhaka, Gaibandha, Jhenaidah, Lalmonirhat, Meherpur, and Savar). The sample was recruited by eight and ten interviewers, respectively. First, the interviewers selected registered users of assistive technology meeting the inclusion criteria. Second, where possible, the interviewers matched each user of assistive technology with the closest living registered person with the same impairment, of the same sex and of similar age (+/− 5 years but not below 15 years). The final sample size was 285 people with hearing impairment and 298 people with ambulatory impairment.

When selecting types of assistive technology to be included in this study, we wanted a variation based on types of represented impairments and required degree of accessibility of the physical environment for efficient use. The main reason for limiting the study to hearing-aids and wheelchairs was that other types of assistive technology were not commonly used or available in Bangladesh. Achieving a reasonable number of respondents using other types of assistive technology to allow for meaningful comparisons was beyond available time and budget frames.

### Instrumentation

The questionnaire used for collecting data consisted of seven parts: demographics, human rights, economy, participation, disability, environment, and assistive technology. Only users of assistive technology answered the last part. The questions were partly based on the International Classification of Functioning, Disability and Health (ICF), a WHO questionnaire [30] and a questionnaire used in livelihood studies in Africa [31], and partly developed by the authors. (see principal outcome questions in Additional file 2: Appendix 2).

### Procedure

The questionnaire was developed in English and translated into Bangla. The translation was reviewed by native and non-native speakers of Bangla, including an expert on communication in simple Bangla. After revision, the questionnaire was pre-tested on 30 people representing various respondent groups followed by a minor revision.

An instruction manual for interviewers was developed and ten interviewers were recruited. All interviewers worked with rehabilitation of people with disabilities in their respective organization. They participated in a four-day training on interviewing and data collection techniques, including one day of practice interviewing using the questionnaire. Following input from the training, the questionnaire was finalized. Supervised by a coordinator, the interviewers collected data between 6 November 2009 and 1 February 2010.

Verbal interviews based on the questionnaire were conducted at the respondent’s home at a single occasion. To protect confidentiality of data, other family members and neighbours were requested to provide privacy. In interviews, where the interviewer was unable to communicate with a participant, data was collected from a proxy. Chi-square tests revealed a statistically significant difference in proxy reporting between users and non-users of hearing aids, while there were no such difference between users and non-users of wheelchairs. Among non-users and users of hearing aids, 109 (73.2%) and 47 (34.6%), respectively, of the questionnaires were completed with the help of proxies.

### Ethical considerations

As there is no authority in Bangladesh that grants ethical approvals, the University of Dhaka was consulted and their ethical research praxis was followed. Potential participants were informed about the study and invited to participate. Only those giving verbal consent were interviewed. Written informed consent could not be used due to high rate of illiteracy. Respondents could refuse to answer any question or discontinue the interview at any time. No incentives for participation were offered.

### Outcome variables

In this work, self-reported enjoyments of six human rights were studied. The rights to standard of living, health, education and work were selected based on their fundamental importance, and rights related to receiving information and to movement were selected based on their relevance for the included impairments. The relations of these rights to the UDHR, the CRPD and the ICF are indicated in Table 1.

**Table 1:** Variables and their relation to UDHR and CRPD articles and ICF categories

**Variable**	**UDHR**	**CRPD**	**ICF**
**Outcome variables**			
Standard of living	25		(d940)
- Adequate food		- 28	- (d550)
- Safe water		- 28	- (d560)
- Adequate clothes		- 28	- (d540)
- Adequate housing		- 28	- (e155)
Health	25	25	(d940)
a. Necessary medical care			a. (e5800)
b. Physical health			b. (BF, BS)
c. Mental health			c. (BF)
Education	26	24	(d940)
a. Reading ability			a. (d166)
b. Primary education			b. (d820)
c. Participation in school			c. d820
Work	23	27	(d940)
a. Working			a. (d850)
b. Participation in employment			b. d850
Receiving information	19	21, 26	(d940)
a. Listening performance			a. d115
Movement	13		(d940)
a. Participation in using public transportation		a. 18	a. d470
b. Ambulating performance		b. 20	b. d450-d460
**Predictor variables**			
Hearing aid use	19	21, 26	(e1251)
Wheelchair use	13	20, 26	(e1201)
**Confounding variables**			
Sex	1, 2	3, 6	(PF)
Age	1, 2	3, 7	(PF)
Place of living	1, 2	3	(PF)
Economic situation	1, 2	3	(PF)
Physical accessibility to working place		9	(e150, e155)
Duration of use		26	(e1201, e1251)
Listening capacity	19	21, 26	d115
Ambulating capacity	13	20, 26	d450-d460

Categories in brackets were not coded or quantified according to the ICF system.

BF = body functions; BS = body structures; PF = personal factors.

Drawing from UDHR Article 25, the right to a standard of living adequate for health and well-being was measured using a composite scale consisting of three items on how frequently the respondent ate three times a day until full, drank safe water and wore clothes adequate for the weather as indicated on 4-point Likerttype scales ranging from ‘Never’ to ‘Always’, and an item on the adequacy of the house for health as indicated on a 3-point Likert-type scale ranging from ‘Not adequate’ to ‘Adequate’. This standard of living scale had good internal consistency with Cronbach alpha coefficients of 0.83 for respondents with hearing impairments and 0.81 for respondents with ambulatory impairments. If the water item was removed, Cronbach alpha would increase by 0.022 and 0.008, respectively. However, as safe water was considered an integral part of this human right, and Cronbach alpha already was satisfactorily high, this item remained.

Three items were used to measure the right to health: medical care, indicating frequency of getting necessary medical care using a 4-point Likert-type scale ranging from ‘Never’ to ‘Always’; and physical health and mental health as indicated on 5-point Likert-type scales ranging from ‘Very bad’ to ‘Very good’.

The right to education was measured using three items: reading ability, which is the ability to read a letter answered by yes or no; primary education, which indicates completion of primary education (i.e. class 5); and participation in school education measured on a 5-point Likert-type scale ranging from ‘Complete problem’ to ‘No problem’.

The right to work was measured by the item work, indicating whether the respondent worked (including being a housewife), and the item participation in employment, which uses an ICF based 5-point Likert-type scale ranging from ‘Complete problem’ to ‘No problem’.

Two items were used to measure freedom of movement: participation in using public transportation and ambulating performance, both using an ICF based 5-point Likert-type scale ranging from ‘Complete problem’ to ‘No problem’.

Freedom to receive information was measured according to the ICF as listening performance using a 5-point Likert-type scale ranging from ‘Complete problem’ to ‘No problem’.

Continuous outcome variables were dichotomized to allow for logistic regression. Due to non-negligible variations in scoring between the hearing and ambulation groups, the dichotomization points were placed at different levels for six of the nine variables, see Table 2. This was done in order to reduce the risk for overfitting [32].

**Table 2:** Dichotomization points of continuous outcome variables for hearing and ambulation groups

**Outcome**	**Hearing**	**Ambulation**
Standard of living		
High	12-15	*Same as hearing*
Low	4-11	
Necessary medical care		
Often	Always *or* Most of the time	*Same as hearing*
Seldom	Seldom *or* Never	
Physical health		
Good	Good *or* Very good	Moderate, Good *or* Very good
Poor	Moderate, Bad *or* Very bad	Bad *or* Very bad
Mental health		
Good	Good *or* Very good	Moderate, Good *or* Very good
Poor	Moderate, Bad *or* Very bad	Bad *or* Very bad
Participation in school		
High	No *or* Mild problem	No, Mild *or* Moderate problem
Low	Moderate, Severe *or* Complete problem	Severe *or* Complete problem
Participation in employment		
High	No, Mild *or* Moderate problem	*Same as hearing*
Low	Severe *or* Complete problem	
Listening performance		
Good	No, Mild *or* Moderate problem	No *or* Mild problem Moderate
Poor	Severe *or* Complete problem	Severe *or* Complete problem
Participation in using public transportation		
High	No *or* Mild problem	No, Mild *or* Moderate problem
Low	Moderate, Severe *or* Complete problem	Severe *or* Complete problem
Ambulatory performance		
Good	No *or* Mild problem	No, Mild *or* Moderate problem
Poor	Moderate, Severe *or* Complete problem	Severe *or* Complete problem

### Predictor variables

The predictor variable for people with hearing impairments, hearing aid user, indicates whether a respondent uses hearing aid(s) or not. Similarly, wheelchair user indicates whether a respondent with ambulatory impairment uses wheelchair or not. In a complementary analysis, duration of use dichotomized into ‘Short’ (less than 3 years) and ‘Long’ (3 years or more) was used as a predictor variable.

### Potential confounding variables

Self-reported enjoyment of human rights was analyzed with respect to reported, possible confounding variables, including sex, age, place of living, and economic situation [33-37]. To determine place of living, the two categories ‘village’ and ‘town/city’ were used. To measure the economic situation, the perception of how the respondent’s household managed economically during the past year was indicated on a self-reported 4-point Likert-type scale ranging from ‘Poorly’ to ‘Very well’.

In complementary analysis, the listening capacity and ambulatory capacity were included as possible confounders of outcomes related to receiving information and movement. Further, it was hypothesized that physical accessibility to the working place was associated with a wheelchair user working or not. Listening capacity and ambulatory capacity were measured as self-reported level of difficulty to listen or to walk or move around without assistance (i.e. without support from assistive technology, other persons, etc.) indicated on an ICF based 5-point Likert-type scale ranging from ‘Unable’ to ‘No difficulty’. Physical accessibility to the working place was self-rated as ‘Good’ or ‘Poor’.

Standard of living may determine health, and education may determine both health and standard of living. However, as we were interested in standard of living and education as outcomes, they were not included as possible causes of other studied effects.

### Analyses

Questionnaire responses were recorded in a Microsoft Access database and analyzed using Statistical Package for Social Sciences (SPSS) version 17.0 statistical software. The analysis was carried out at three levels. First, descriptive statistics and t-tests, Mann–Whitney *U* test, and Pearson’s chi-square test were used to report on differences in profile characteristics between respondent groups. Second, crude odds ratios (OR) and 95% confidence intervals (95% CI) were calculated to explore associations between assistive technology use and the outcome variables, which were dichotomized if not already binary. Third, analysis by logistic regression was performed to investigate the potential importance of various confounders and to analyze whether use of assistive technology can predict differences in enjoyment of human rights. To avoid overfitting, i.e. less than 10–15 events per predictor and confounding variables [32], adjustments were not made for participation in school, participation in work, and listening performance among respondents with ambulatory impairment. To indicate the potential importance of accessibility for participation in work among people with ambulatory impairments, an over fitted analysis is presented in table 5 (8.5 events per variable adjusted for). Chi-square tests were done to assess the impact of proxies answering the questions on behalf of the respondents.

## Results

### Respondent characteristics

Characteristics of the respondents are given in Table 3, including their self-rated listening and ambulatory capacities. There were statistically significant differences between the groups of users and non-users of hearing aids in terms of mean age, economic situation and place of living, while no such differences were found between the groups of users and non-users of wheelchairs. None of the non-users of wheelchairs possessed a mobility device that they claimed using.

**Table 3:** Characteristics of non-users and users of hearing aids and wheelchairs

**Characteristic**	**Non-users**	**Users**	***p*-value**
Respondents with hearing impairment	N = 149	N = 136	
	*Mean ± SD*	*Mean ± SD*	
Age (years)	30.4 ± 11.6	26.5 ± 13.3	0.010
Economic situation (1–4)	1.58 ± 0.71	2.00 ± 0.91	<0.001
Listening capacity (1–5)	1.89 ± 0.69	2.03 ± 0.68	0.078
Ambulatory capacity (1–5)	4.28 ± 1.06	4.28 ± 1.11	0.770
Duration of use (years)	-	5.7 ± 4.2	-
	*n (%)*	*n (%)*	
Sex (Man)	83 (55.7)	85 (62.5)	0.296
Place of living (Village)	126 (84.6)	88 (64.7)	<0.001
Respondents with ambulatory impairment	N = 149	N = 149	
	*Mean ± SD*	*Mean ± SD*	
Age (years)	32.1 ± 12.4	31.8 ± 13.1	0.853
Economic situation (1–4)	1.52 ± 0.65	1.70 ± 0.78	0.057
Listening capacity (1–5)	4.62 ± 0.91	4.64 ± 0.96	0.596
Ambulatory capacity (1–5)	2.07 ± 0.76	2.00 ± 0.71	0.501
Duration of use (years)	-	4.5 ± 3.7	-
	*n (%)*	*n (%)*	
Sex (Man)	95 (63.8)	110 (73.8)	0.080
Place of living (Village)	119 (79.9)	106 (71.1)	0.106

SD = Standard deviation.

### Crude odds ratios

Distribution of dichotomized outcomes by respondent category is presented in part A of Table 4. Crude Odds Ratios for studied outcomes for users of assistive technology compared to non-users are provided in part B of Table 4. All associations were statistically significant among respondents with hearing impairments. And among respondents with ambulatory impairments the two associations between wheelchair use and necessary medical care and ambulatory performance, respectively, were statistically significant.

**Table 4:** Distribution of dichotomized outcomes (A), crude odds ratios (B), and adjusted odds ratios (C)

**Outcome:**	**High standard of living**	**Often necessary medical care**	**Good physical health**	**Good mental health**	**Can read a letter**	**Completed primary education**	**High participation in school**	**Work (incl. housewife)**	**High participation in work**	**Good listening performance**	**High participation in using public transportation**	**Good ambulatory performance**
*A. Distribution of dichotomized outcomes, % (n)*												
Users of hearing aids	68.9 (93)	70.4 (95)	50.7 (69)	44.9 (61)	67.6 (92)	58.1 (79)	60.3 (35)	47.1 (64)	52.4 (22)	75.0 (102)	64.2 (86)	94.8 (128)
Non-users of hearing aids	42.6 (63)	43.9 (65)	30.2 (45)	23.5 (35)	25.5 (38)	22.8 (34)	33.3 (8)	69.8 (104)	14.7 (5)	20.3 (30)	39.6 (59)	72.1 (106)
Users of wheelchairs	48.3 (72)	48.0 (71)	63.1 (94)	54.4 (81)	47.0 (70)	39.6 (59)	55.2 (16)	31.5 (47)	42.4 (14)	90.6 (135)	31.7 (45)	52.3 (78)
Non-users of wheelchairs	38.9 (58)	35.8 (53)	53.7 (80)	45.6 (68)	38.9 (58)	36.2 (54)	45.7 (16)	40.9 (61)	37.1 (13)	86.6 (129)	31.7 (46)	26.8 (40)
*B. Crude Odds Ratios (95% CI) regarding use of assistive technology versus no use*												
Use of hearing aid	**3.00** (1.83-4.87)	**3.03** (1.86-4.96)	**2.38** (1.46-3.87)	**2.65** (1.60-4.40)	**6.11** (3.65-10.2)	**4.69** (2.81-7.82)	**3.04** (1.12-8.26)	**0.38** (0.24-0.62)	**6.38** (2.07-19.7)	**11.8** (6.75-20.6)	**2.73** (1.69-4.43)	**7.07** (3.05-16.4)
Use of wheelchair	1.47 (0.93-2.32)	**1.65** (1.04-2.63)	1.47 (0.93-2.34)	1.42 (0.90-2.24)	1.39 (0.88-2.20)	1.15 (0.72-1.84)	1.46 (0.54-3.93)	0.66 (0.41-1.07)	1.25 (0.47-3.30)	1.50 (0.72-3.08)	1.00 (0.61-1.64)	**3.00** (1.84-4.86)
*C. Adjusted Odds Ratios (95% CI) regarding use of assistive technology versus no use*												
Use of hearing aid	**2.07** (1.17-3.65)	**1.82** (1.003-3.32)	**1.80** (1.07-3.02)	**2.04** (1.19-3.50)	**5.05** (2.90-8.78)	**3.84** (2.19-6.71)	**5.16** (1.57-17.0)	0.56 (0.31-1.02)	**4.94** (1.31-18.6)	**13.6** (7.28-25.5)	**2.06** (1.23-3.44)	**6.06** (2.52-14.6)
Use of wheelchair	1.28 (0.76-2.16)	1.33 (0.75-2.36)	1.21 (0.74-1.99)	1.23 (0.76-1.97)	1.11 (0.67-1.84)	0.88 (0.53-1.48)	1.18 (0.38-3.60)	**0.59** (0.36-0.98)	0.65 (0.20-2.13)	-	0.85 (0.50-1.42)	**3.04** (1.84-5.01)

Adjustments were made for sex, age, place of living and economic situation. (Bold = Significance level at p<0.05).

CI = Confidence Interval.

### Adjusted odds ratios

Adjusted Odds Ratios regarding use versus no use of hearing aids and wheelchairs after adjusting for sex, age, place of living and economic situation are presented in part C of Table 4.

People using hearing aids were more likely to report a high standard of living, OR = 2.1 (1.2-3.7), that they often receive necessary medical care, OR = 1.8 (1.0-3.3), good physical health, OR = 1.8 (1.1-3.0), and good mental health, OR = 2.0 (1.2-3.5). Although wheelchair users also tended to score higher on these outcomes compared to non-users, the differences were not significant.

Compared to non-users of hearing aids, users were more likely to report that they can read, OR = 5.0 (2.9-8.8), have completed primary education, OR = 3.8 (2.2-6.7), and have a high participation in school, OR = 5.2 (1.6-17.0). Among users and non-users of wheelchairs, there were no significant differences in educational outcomes.

Respondents using hearing aids were more likely to report a high level of participation in work compared to non-users, OR = 4.9 (1.3-18.6), while the difference in reported work status was not significant. Compared to non-users of s, users were less likely to report that they work, OR = 0.59 (0.36-0.98). The difference in reported work status between hearing aid users and non-users was not statistically significant.

Hearing aid users were much more likely to report good listening performance compared to non users, OR = 13.6 (7.3-25.5). Users of hearing aids were also more likely to report high participation in using public transportation, OR = 2.1 (1.2-3.4), and more likely to report good ambulatory performance compared to non-users, OR = 6.1 (2.5-14.6). There were no statistically significant differences between users and non-users of wheelchairs in terms of participation in using public transportation, while users were more likely to report good ambulatory performance, OR = 3.0 (1.8-5.0).

Odds Ratios for work related outcomes among users and non-users of wheelchairs after adjusting for physical accessibility to the working place are given in Table 5. There was no statistically significant difference between users or non-users of wheelchairs in reported working status. Compared to non-users, wheelchair users reported a higher degree of participation in work, OR = 6.7 (1.2-37.5). Table 5 also indicates that respondents that had had a wheelchair for three years or more were more likely to report that they work compared to those who had had a wheelchair for a shorter period of time, OR = 3.5 (1.6-7.8).

**Table 5:** Odds ratios (95% CI) regarding effects of wheelchair use on work related outcomes. (Bold = Significance level at p < 0.05)

**Predictor variable**	**Work (incl. housewife)**	**High participation in work**
Use of wheelchair (vs. No use)^a^	0.75 (0.38-1.47)	**6.68** (1.19-37.5)
Long duration of use of wheelchair (vs. Short)	**3.49** (1.57-7.76)	**-**

^a^ Adjusted for level physical accessibility to the working place.

Adjustments for listening capacity and ambulatory capacity yielded higher odds ratios regarding assistive technology use versus non-use for corresponding performances.

### Proxy responses

Chi-square tests of outcomes of respondents with hearing impairments between respondent reported and proxy reported data revealed significant differences for 3 of the 12 outcomes. Significantly less (p < 0.001) proxies indicated that the respondent can read a letter, has completed primary school, and has good listening performance. Although not statistically significant, proxies tended to report better physical health than respondents themselves (p = 0.065).

## Discussion

In an attempt to explore the relation between assistive technology use and human rights enjoyment in a low-income country, cross-sectional data from users and non-users of hearing aids and wheelchairs in Bangladesh was analyzed using logistic regression. The findings provide empirical support for using assistive technology interventions to facilitate full enjoyment of human rights. The results complement a previous report from the same sample, which found that assistive technology use is associated with better opportunities to do things one has reason to value and with better attitudes from neighbours [38].

### Use of hearing aids

Apart from non-significant differences in working status, people using hearing aids were more likely to report positive outcomes regarding standard of living, health, education, work, receiving information, and movement than people with hearing impairments not using hearing aids did. The results support the general positive outcomes of hearing aid use on activities and participation found in Brazil and Nigeria [39,40]. A study in India reported that regular hearing aid use had a positive impact on the performance of students, particularly on language [4], which may partly explain the positive relation between hearing aid use and education outcomes reported in this paper.

The non-significant differences in working status between users and non-users of hearing aids are consistent with findings in Australia [41]. However, they call for a deeper analysis which was not possible with the available data. Such an analysis may consider including attitudes of employers and aspects of the labour market.

We did not anticipate that hearing aid use would be positively related to the enjoyment of freedom of movement in terms of ambulatory performance and participation in using public transportation. However, in an environment with little visual instructions the positive outcome of hearing aid use on listening performance may explain this, as much of the readily available or sought after information for movement and risk avoidance are audible in Bangladesh. In addition, where instructions and information are available in written, users of hearing aids are likely to benefit as they were literate to a greater extent than non-users. If further studies confirm the positive association between hearing aid use and the enjoyment of the freedom of movement, the possibility of including such devices as assistive technology for personal mobility in accordance with the CRPD may be considered. The CRPD explicitly requires governments to make such technology available at affordable cost [2].

### Use of wheelchairs

Few significant differences between users and non-users of wheelchairs were found. Wheelchairs do fulfil their intended purpose of providing mobility, which correspond to reported outcomes in India and Peru [3]. On the contrary, the lack of significant differences in physical and mental health contrast with findings from India and Peru, where nearly 50% of the respondents reported that health had improved after receiving a wheelchair [3]. Based on findings in Uganda, it has been assumed that assistive technology for mobility would provide opportunities for education and employment [42]. However, this study and the study in India and Peru indicate that such opportunities are not directly materialized as no significant differences due to wheelchair use alone were found [3]. The negative association between wheelchair use and working status called for a complementary analysis, which indicated that physical accessibility and duration of use have an effect on working status. The statistically significant association between physical accessibility and high participation in work need to be interpreted cautiously as the analysis was overfitted (8.5 events per variable adjusted for). However, the findings support current guidelines for provision of wheelchairs, where assessment of physical accessibility of intended environments of use is recommended [25]. Assessing and ensuring physical accessibility appears a prerequisite for users to be able to benefit from a wheelchair for other human rights purposes than movement. However, it is uncertain if the degree of physical accessibility fully explains why wheelchair users are not more likely to report positive outcomes than non-users do, as there are more aspects of the environment which may dynamically influence participation [43,44]. As for the hearing group, further investigations are required to explore factors which impact these outcomes.

### Limitations

There have been earlier attempts to quantitatively assess the field of health and human rights [45]. However, to our knowledge, this is the first reported study focusing specifically on the relation between assistive technology use and human rights outcomes, which left us with little guidance on how to approach the work. We are aware that the study has several limitations which should be considered when interpreting the findings.

An inherent limitation of a cross-sectional design is its inapplicability in exploring cause and effect relationships. Longitudinal studies are needed to assess the role assistive technologies play in the link between impairments and human rights enjoyment.

Like most countries, Bangladesh does not maintain a national register of users of assistive technology, and as the prevalence of assistive technology use is very low, it was impossible to achieve a representative sample within the resource constraints of this research. It is often difficult to obtain representative samples in low-income countries, particularly when hidden and vulnerable population groups are involved [46,47]. Therefore, as the sample in this study was not randomly selected, there is a risk for selection bias, and thus we must be cautious about generalizing the findings to all people with hearing and ambulatory impairments in Bangladesh. It can be noted that in all sampling areas all registered and eligible users of hearing aids and wheelchairs, respectively, were included. As there were more non-users of assistive technology in the areas, each user of assistive technology was matched with a non-user as far as the circumstances allowed. Besides this, there was no obvious difference in the chance to be selected because of use of assistive technology or not. As indicated in table 3, no statistically significant differences of key-characteristics between users and non-users of wheelchairs were found, while such differences occurred between users and non-users of hearing aids for age, place of living and economic situation.

The hearing and ambulatory capacities of the respondents was not assessed by a trained professional. In case the capacity of non-users of assistive technology would exceed the capacity of users, it is likely that any differences in outcomes would have been underestimated given that assistive technology benefits its user. On the contrary, if the capacity of users exceeds the capacity of non-users, it is likely that any differences in outcomes would have been overestimated. Although no statistically significant differences in self-rated capacities between users and non-users of assistive technology were found, the lack of objective assessment of the respondents’ capacities constitutes a limitation of the study.

People associated with a development organization may have had the opportunity to be empowered to exercise their human rights to a larger extent than people that are not associated with such an organization. It is therefore likely that our sample would score higher compared to people who would not be associated with an organization. However, our sample could well score lower than those who are associated with other organizations. As this work was limited to studying the relative difference between users and non-users of assistive technology living in the same communities and associated with the same organizations, we assume that this has not had a significant effect on the result.

The use of an administered questionnaire can result in systematically biased answers as responses may be given to satisfy the interviewer, but as we only compare data provided by respondents within this single country context, such bias may not significantly affect the conclusions. Further, we relied on self- and proxy-reported data and do not know how closely the responses correlate with objective measures. Understanding of Likert-type scales may vary, which may influence individual responses.

Existing variations between self- and proxy-reported data seems reasonable, i.e. that the reason for a proxy answering may be that the respondent’s listening performance is poorer, resulting in lower completion of primary education, and, consequently, the respondent remaining unable to read. Thus, as the variations in reported outcomes between respondents and proxies seem logical, it appears that the proxy-reported data can be considered reliable.

For the purpose of simplicity in presenting the logistic regression models in parts C of Table 4, and given the limitations of the sample size, adjustments were made for the same confounders for all outcomes. This resulted in including confounders whose correlation with some outcomes had not necessarily been previously reported and excluding known confounders. This could potentially affect the findings. However, preliminary adjustments of reported confounders only marginally affected the results. As all dichotomization points are not the same for the hearing and ambulatory groups, it is not possible to compare these groups for all outcomes.

## Conclusions

Use of hearing aids and wheelchairs was positively associated with human rights enjoyment in Bangladesh. With an exception for non-significant differences in work status, users of hearing aids were more likely to report high levels of enjoyment of all studied human rights compared to non-users. Wheelchair users were likely to report less difficulty to move around compared to non-users, and, after adjusting for physical accessibility to the working place, more likely to enjoy the right to work. This underscores the necessity of considering physical accessibility in wheelchair services. Further studies are needed to understand what factors affect the relation between work status and assistive technology use in low-income countries in order to find strategies to facilitate work. Longitudinal studies are required to assess the temporal association between assistive technology use and human rights enjoyment.

## Abbreviations

CDD: Centre for Disability in Development; CRPD: Convention on the Rights of Persons with Disabilities; GDP: Gross Domestic Product; ICF: International Classification of Functioning Disability and Health; PPP: Purchasing Power Parity; Standard Rules: Standard Rules on the Equalization of Opportunities for Persons with Disabilities; UDHR: Universal Declaration of Human Rights; WHO: World Health Organization.

## Competing interests

JB, SL, POÖ and ASMAR declare that they have no competing interests. NB and AHMNK work in a non-governmental organization whose activities include provision of assistive technology. However, they have not been involved in the analysis and interpretation of data.

## Authors’ contributions

JB conceived of the study, participated in its design, performed the statistical analysis, and drafted the manuscript. SL conceived of the study, participated in its design, and helped to draft the manuscript. POÖ participated in the design of the study, helped to perform the statistical analysis, and helped to draft the manuscript. ASMAR participated in the design of the study, helped in acquisition of data, and helped to draft the manuscript. NB participated in the design of the study, supervised acquisition of data, and helped to draft the manuscript. AHMNK participated in the design of the study, helped in acquisition of data, and helped to draft the manuscript. All authors read and approved the final manuscript.

## Pre-publication history

The pre-publication history for this paper can be accessed here:

http://www.biomedcentral.com/1472-698X/12/18/prepub

## Supplementary Material

Additional file 1 **Appendix 1.** Power protocol. Click here for file

Additional file 2 **Appendix 2.** Questions for principal outcomes. Click here for file
