# Supplementary material for: Assistive technology use and human rights enjoyment: a cross-sectional study in Bangladesh
Source: BMC Int Health Hum Rights. 2012 Sep 19;12:18. doi: 10.1186/1472-698X-12-18 (PMC3519502; doi:10.1186/1472-698X-12-18)

**Appendix 1. Power protocol**

The power calculation was performed using G*Power 3.1.2 with the input parameter values indicated below.


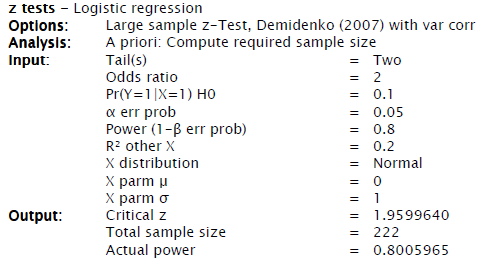

Supplement: Additional file 1 — Appendix 1. Power protocol. [file 1472-698X-12-18-S1.doc]
