# Supplementary material for: Assistive technology use and human rights enjoyment: a cross-sectional study in Bangladesh
Source: BMC Int Health Hum Rights. 2012 Sep 19;12:18. doi: 10.1186/1472-698X-12-18 (PMC3519502; doi:10.1186/1472-698X-12-18)
Supplement: Additional file 2 — Appendix 2. Questions for principal outcomes. [file 1472-698X-12-18-S2.doc]

**Appendix 2. Questions for principal outcomes**

Only those items of the questionnaire that were used as principal outcome questions in this particular study are reproduced below. Therefore, the numbering of the items may seem inconsistent.

| 1.1.1 **Do you eat food three times a day?** | | | | | | | | | | | | | |
| --- | --- | --- | --- | --- | --- | --- | --- | --- | --- | --- | --- | --- | --- |
| □ Always | | | □ Most of the time | | | □ Seldom | | | | □ Never | | | |
| 1.1.2 **Do you regularly drink safe water?** | | | | | | | | | | | | | |
| □ Always | | | □ Most of the time | | | □ Seldom | | | | □ Never | | | |
| 1.1.3 **Do you wear clothes which are appropriate for the weather?** | | | | | | | | | | | | | |
| □ Always | | | □ Most of the time | | | □ Seldom | | | | □ Never | | | |
| 1.1.4 **Do you get necessary medical care?** | | | | | | | | | | | | | |
| □ Always | | | □ Most of the time | | | □ Seldom | | | | □ Never | | | |
| 1.1.5 **Do you live in a house which is adequate for your health?** | | | | | | | | | | | | | |
| □ Adequate | | | | □ Reasonably adequate | | | | | | □ Not adequate | | | |
| 1.4.1 **Can you read a letter?** | | | | | | | | | □ Yes | | | | □ No |
| 1.4.3 **Have you ever studied in school?** | | | | | | | | | □ Yes | | | | □ No |
| 1.4.3.1 If **Yes**, up to which class did you complete studies in school? | | | | | | | | | | | | |  |
| 1.5.1 **Are you currently working?** (includes waged work and self-employment) | | | | | | | | | | | | | |
| □ Yes | □ No, but have been working before | | | | | | □ No, never worked | | | | □ I am a housewife | | |
| 1.6.1 **How would you describe your overall physical health today considering sickness, illness, injury, disease, etc.?** | | | | | | | | | | | | | |
| □ Very good | | □ Good | | | □ Moderate | | | □ Bad | | | | □ Very bad | |
| 1.6.2 **How would you describe your overall mental health today considering anxiety, fear, fatigue, tiredness, hopelessness, etc.?** | | | | | | | | | | | | | |
| □ Very good | | □ Good | | | □ Moderate | | | □ Bad | | | | □ Very bad | |

| 3.1 **I will now ask questions about how much problem you may have to carry out activities in your current environment, which is where you spend most of your time, for example home, work and school. It includes assistance from other people and the use of assistive devices.** | | | | |
| --- | --- | --- | --- | --- |
| 3.1.1 **To what extent do you have problem to use transportation as a passenger to move around?** | | | | |
| □ No problem  □ Mild problem | | □ Moderate problem  □ Severe problem | | □ Complete problem  □ Not applicable |
| 3.1.10 **To what extent do you have problem to go to school and study?** | | | | |
| □ No problem  □ Mild problem | | □ Moderate problem  □ Severe problem | | □ Complete problem  □ Not applicable |
| 3.1.11 **To what extent do you have problem to get and keep a job?** | | | | |
| □ No problem  □ Mild problem | | □ Moderate problem  □ Severe problem | | □ Complete problem  □ Not applicable |
| 4.5 **Please indicate the extent to which you are able to participate in the following activities IN YOUR CURRENT ENVIRONMENT, which is where you spend most of your time, for example home, work and school. This may include assistance from other people, the use of assistive devices, etc.** | | | | |
| 4.5.1 **To what extent do you have problem to listen in your current environment?** | | | | |
| □ No problem  □ Mild problem | □ Moderate problem  □ Severe problem | | □ Complete problem | |
| 4.5.6 **To what extent do you have problem to move around in your current environment?** | | | | |
| □ No problem  □ Mild problem | □ Moderate problem  □ Severe problem | | □ Complete problem | |
